# Delayed Diagnosis of West Nile Meningoencephalitis in a Patient Receiving Rituximab for Rheumatoid Arthritis

**DOI:** 10.7759/cureus.30221

**Published:** 2022-10-12

**Authors:** Abdelmohaymin A Abdalla, Joseph Fanciullo, Huthayfa Ateeli

**Affiliations:** 1 Pulmonary and Critical Care Medicine, Mayo Clinic, Phoenix, USA; 2 Rheumatology, University of South Dakota, Sioux Falls, USA; 3 Pulmonary Critical Care, Avera McKennan Hospital and University Health Center, Sioux Falls, USA

**Keywords:** rheumatoid arthritis, rituximab, hypogammaglobulinemia, encephalitis, wnv

## Abstract

West Nile virus (WNV) neuroinvasive disease is associated with substantial morbidity and mortality. Clinical suspicion is usually confirmed with cerebrospinal (CSF) immunoglobulin M (IgM) detection using enzyme-linked immunoassay (ELISA) techniques. CSF polymerase chain reaction (PCR) is rarely used to confirm the disease and is not widely available. We present a detailed report of false-negative WNV IgM in a patient receiving rituximab therapy for rheumatoid arthritis. She was exposed to the virus during peak immunosuppression and strong clinical suspicion was confirmed with WNV PCR, illustrating the importance of such consideration with the recent incremental use of rituximab therapy. Despite the lack of specific anti-viral treatment for WNV, delayed consideration and diagnosis of WNV in those who are immunosuppressed would expose them to a wide panel of testing, with a subsequent increase in the cost of medical care.

## Introduction

West Nile virus (WNV) has been linked to many forms of neuroinvasive diseases, including meningitis, encephalitis, and myelitis [1]. Disease onset for most of the cases was reported to occur between July and September [2]. Clinical diagnosis is mostly confirmed with the detection of immunoglobulin (Ig) M against WNV in the serum or cerebrospinal fluid (CSF) [3]. Nonspecific CSF findings include pleocytosis and elevated protein [2-4]. Although viral detection using polymerase chain reaction (PCR) is highly specific, it’s not available in many commercial laboratories and the test is usually sent to reference laboratories [5,6]. There are no guidelines that address the diagnostic challenges in immunosuppressed patients. In this report we present an unfortunately delayed diagnosis of progressive WNV encephalitis in a returning traveler, focusing on diagnostic challenges and test performances. The objective of the study is to provide more insights through detailed case descriptions into the diagnostic certainty issues of Ig testing in the setting of immunosuppression.

## Case presentation

A 62-year-old Caucasian female was seen in the emergency department in September following a seven-day history of non-specific symptoms, including malaise, nausea, headache, and fever. Her symptoms progressed despite over-the-counter acetaminophen. A day prior to presentation, she visited her primary care physician, who recommended supportive care and bed rest. However, she became increasingly confused and, ultimately, her family brought her to seek urgent medical care. She had no abdominal pain, diarrhea, or changes in her bowel habits. A review of systems was negative for cardiopulmonary and genitourinary symptoms. She complained of no focal weakness and had no cranial nerve symptoms. She had no new skin rash.

A few days prior to presentation, she came back from a hiking trip to Peru, where she visited Machu Picchu, near the Andes mountains. Though the exact altitude she climbed is unknown, she was able to perform all activities as usual, including climbing, with no cardiopulmonary symptoms. Of note, she was trying to keep her utmost fitness prior to the trip. There were no known sick contacts before, during, or after the trip. Her past medical history was significant for long-standing seropositive rheumatoid arthritis that has been well-controlled with rituximab therapy. She received the last dose of rituximab eight weeks prior to presentation. She never smoked cigarettes and drank alcohol occasionally. She had a known severe allergy to penicillin.

On clinical examination, the patient was ill-appearing and lethargic. Her blood pressure was 140/90 mmHg with a temperature of 103 F. Her pupils were equally reactive bilaterally. She had no neck rigidity. Neurological examination showed no focal deficit, but there was generalized weakness and areflexia. The remainder of the examination was normal, including her skin, cardiopulmonary system, and abdomen. 

Complete blood count showed a white blood cell (WBC) count of 7.8 10^9^L and hemoglobin of 9.8 g/dl with normal mean corpuscular volume. Blood chemistry, including serum glucose, was normal. Blood cultures were collected at the time of admission. and they were negative. Influenzas A and B testing were negative, as well as human immunodeficiency virus and syphilis screening. Thick and thin blood films for malaria were negative.

**Figure 1:**
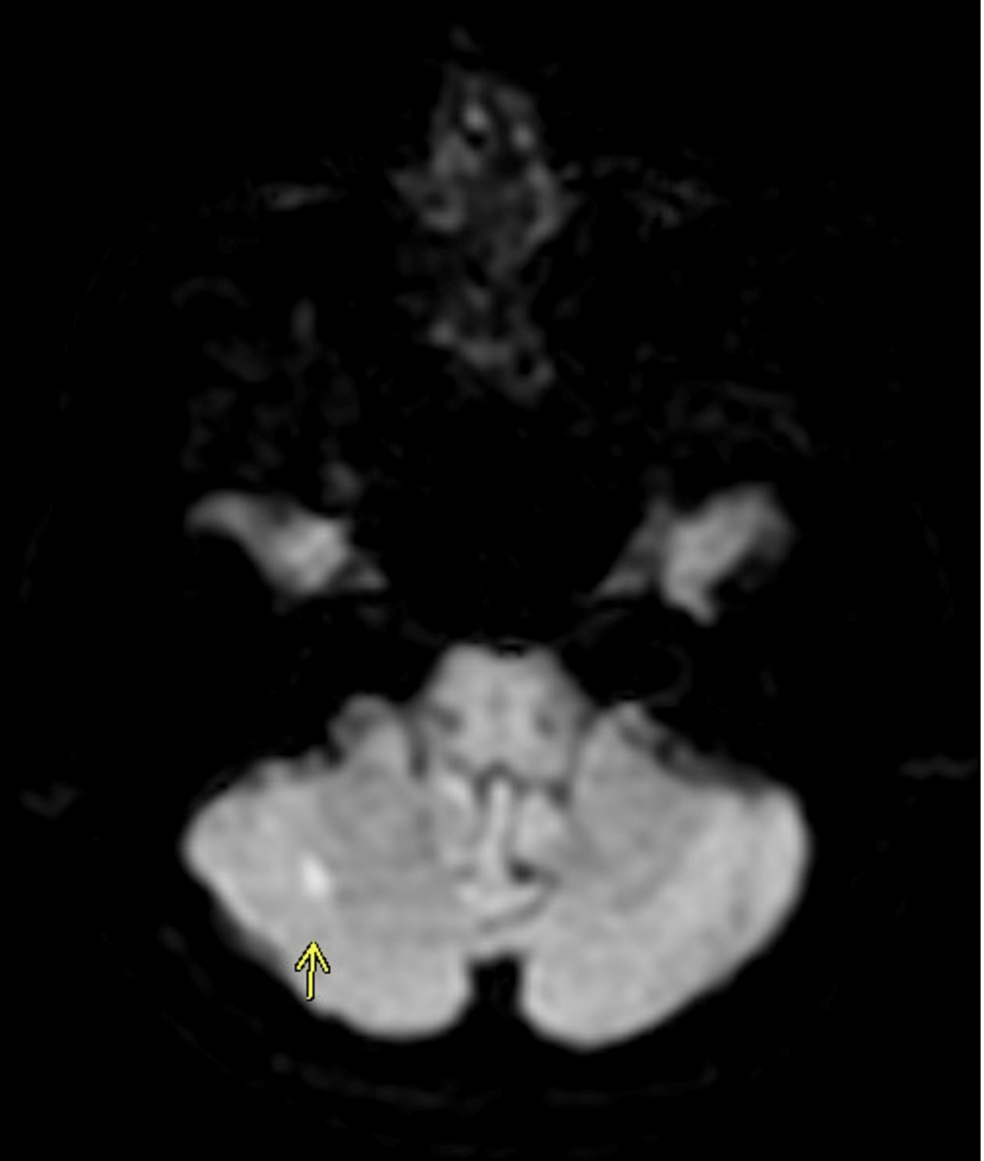
Diffusion-weighted magnetic resonance imaging (DWI-MRI) showing cerebellar infarct (arrow)

**Figure 2:**
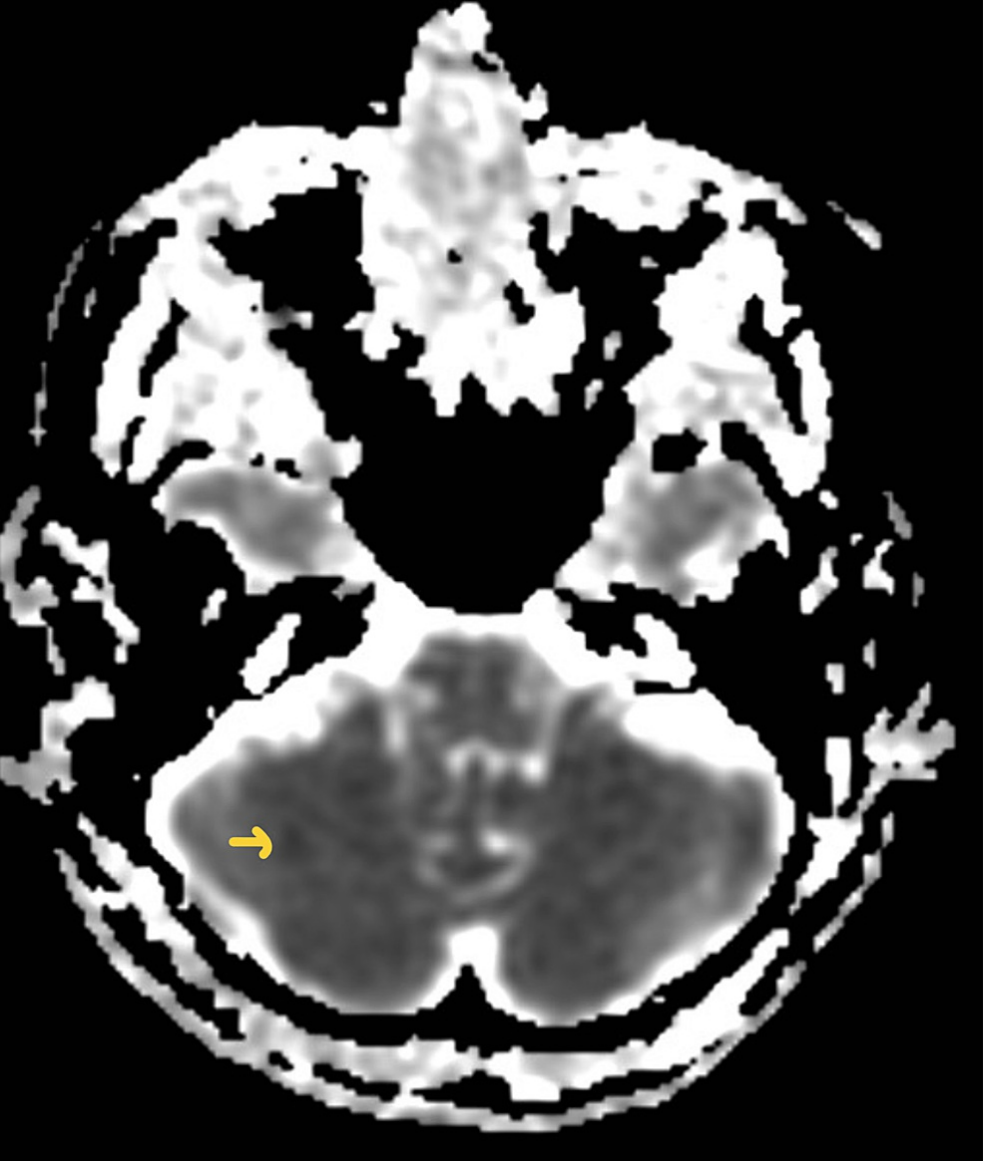
Magnetic resonance imaging with apparent coefficient mapping (MRI-ADC) showing cerebellar infarct (arrow)

The patient was admitted to the hospital and her mental status continued to decline, requiring endotracheal intubation for airway protection and hypoxemia. Computed tomography of the head showed no acute abnormality. Later that day, magnetic resonance Imaging of the brain showed an acute right-sided cerebellar stroke (Figures 1, 2).

A lumbar puncture was performed. The opening pressure was 28 cmH2O. CSF was colorless, containing WBCs (99 cells/mm) and RBCs (11 cells/mm). The results of the differential WBC are as follows: 2% neutrophils, 57% lymphocytes, and 41% monocytes. Glucose was 63 mg/dl and total protein was high at 106 mg/dl. Lactate dehydrogenase was 36 mg/dl. Gram stain and culture were negative for bacteria, including anaerobes.

The patient received broad-spectrum antimicrobial therapy, including antivirals. Infectious team consultation was warranted. CSF and serum serologies were extended to include serologies for cryptococcus antigen, Lyme IgG/IgM, Zika virus IgM, West Nile IgG/IgM, Strongyloides IgG, Varicella zoster, and toxoplasma IgG/IgM. All of them came back negative.

Ig levels were checked and were low. IgG was 392 L, IgA was 72 L and IgM was 17 L. IgG was checked a week prior to critical illness and it was 624 L. CSF polymerase chain reaction (PCR) was thereafter obtained for EBV, CMV, E.coli, Herpes type I, herpes type II, HHV 6, listeria, Hemophilus influenza, Streptococcus pneumonia/agalactiae, VZV, and enterovirus. All of them were also negative. A CSF sample was sent for West Nile virus PCR to the Centers for Disease Control and Prevention (CDC).

The patient continued to spike low-grade fevers, in the range of 100.6-100.9 F. Her mental status was fluctuating, being remarkably different on each clinical encounter. She failed an extubation attempt along with multiple spontaneous breathing trials due to poor mentation and generalized weakness. Two weeks after her admission, her family agreed to proceed with tracheostomy/percutaneous gastric tube placement with plans of long-term weaning from mechanical ventilation, if she was to improve. At that time, West Nile Virus CSF PCR was still pending and, essentially, there was no definite diagnosis at hand. This put extra stress on her family members and on the treating team, let alone the resources utilized and the cost of extra/repeated testing that was non-revealing/repeatedly negative. Six weeks after her admission, the CDC reported a positive result for the WNV PCR from repeat CSF tests.

## Discussion

WNV is considered an endemic disease in North America, with an incubation period ranging from 2 to 21 days [2]. Most patients present with non-specific symptoms, including nausea, headache, muscle pain, and generalized weakness. Clinical neuroinvasive disease with encephalitis has been reported in about 10% of patients with a trend toward more severe disease in cancer patients undergoing chemotherapy and organ transplant recipients [7,8]. Testing for serum and CSF IgM using enzyme-linked Immunosorbent assay (ELISA) is done for suspected cases after initial CSF chemical analysis that typically shows pleocytosis [4]. Those antibodies are usually detectable after three to eight days from the onset of the disease but persist for 30-90 days. Depending on how high the titer is, and whether testing for other arboviruses was concomitantly done, the CDC categorizes those who have positive ELISA IgM as probable or confirmatory. Those with positive PCR for WNV are considered as having a confirmed disease [9]. Clinicians usually resort to more rapid and available testing to confirm the disease, especially if the clinical presentation is non-specific and rapidly evolving.

Rituximab induces the death of B lymphocytes by binding to CD 20. Cellular death occurs within 48 hours after administration, and mature B lymphocyte count remains low for two to six months [10,11]. Patients with exposure to WNV during this peak immunosuppression might be at a higher risk of getting a more severe disease with lethal neuroinvasive infections [12]. As of now, this is the second case of such rapidly worsening neurological illness in patients with rheumatoid arthritis treated with rituximab. Unfortunately, the first case passed away after a more comfortable path was sought [13]. This has been shown in rodent models as well, as infected mice during peak rituximab immunosuppression had more central nervous system (CNS) viral burden and worsened mortality [14]. This puts more emphasis on the importance of obtaining PCR testing for patients who have their disease onset within the peak rituximab effect if WNV infection is suspected on clinical grounds, but rapid ELISA IgM testing is negative.

Being a single-patient study in an endemic area of WNV adds to the inherent limitations of our study. Another technical limitation was the unavailability of PCR in our institution, which could suggest an inter-laboratory observer bias. However, advanced PCR for WNV is rarely available outside CDC in most midwestern states in the US.

## Conclusions

While PCR testing for WNV is not widely available, it might be essential in confirming the clinical suspicion of neuroinvasive disease. This becomes more relevant in patients exposed to the infection during peak rituximab immunosuppression, as it might result in a false-negative ELISA IgM test. Though no specific anti-viral agent has been approved for treating WNV, an early diagnosis might decrease the cost of multiple tests that are routinely done for immunosuppressed patients.
